# Large gastroduodenal artery pseudoaneurysm, arterioportal fistula and portal vein stenosis in chronic pancreatitis treated using combined transarterial embolization and transportal stenting: A case report

**DOI:** 10.1097/MD.0000000000032593

**Published:** 2022-12-30

**Authors:** Sung Gong Lim, Sung Eun Park, In Chul Nam, Ho Cheol Choi, Jung Ho Won, Sa Hong Jo, Hye Jin Baek, Jin Il Moon, Eun Cho, Jae Yool Jang

**Affiliations:** a Department of Radiology, Gyeongsang National University School of Medicine and Gyeongsang National University Changwon Hospital, Changwon, Korea; b Department of Radiology, Jeju National University School of Medicine, Jeju National University Hospital, Jeju, Korea; c Department of Radiology, Gyeongsang National University School of Medicine and Gyeongsang National University Hospital, Jinju, Korea; d Department of General Surgery, Gyeongsang National University School of Medicine and Gyeongsang National University Changwon Hospital, Changwon, Korea.

**Keywords:** arterioportal fistula, case report, gastroduodenal artery pseudoaneurysm, portal vein stenosis, transarterial embolization

## Abstract

Chronic pancreatitis is an ongoing fibroinflammatory disease of the pancreas characterized by irreversible damage to the pancreatic parenchyma and ductal system. Besides, chronic pancreatitis can present with a variety of life-threatening complications.

**Patient concerns:**

The patients visited our hospital due to abdominal pain and anemia, and had chronic pancreatitis as an underlying disease.

**Diagnoses:**

Computed tomography showed a large gastroduodenal artery pseudoaneurysm, arterioportal vein fistula, and portal vein stenosis.

**Interventions:**

We would like to report the successful use of the coils, and N-butyl cyanoacrylate glue for the therapeutic embolization of the pseudoaneurysm and fistula between the gastroduodenal artery and the portal vein, and stenting for portal vein stenosis.

**Outcomes:**

On the day following the endovascular management, the patient reported remission of abdominal pain, and hemoglobin level returned to normal after transfusion. It was confirmed that it was still well maintained in the follow-up examination after 1 month.

**Lessons:**

Although chronic pancreatitis causes many vascular complications, simultaneous occurrence of these lesions is extremely rare. Herein, we share our experience with a unique case of an extrahepatic arterioportal fistula induced by the rupture of gastroduodenal artery pseudoaneurysm with concomitant portal vein stenosis. In these complex cases, combined transarterial embolization and transportal stenting can be helpful.

## 1. Introduction

Visceral artery pseudoaneurysms are relatively rare complications of chronic pancreatitis.^[1]^ The reported incidence of pseudoaneurysm formation from pancreatitis varies from 1.3% to 10% among different case series.^[2]^ Nevertheless, chronic pancreatitis is one of the most common causes of such pseudoaneurysm.^[3]^ with the splenic artery being the most frequently involved (50%), followed by the gastroduodenal (10%–15%), pancreaticoduodenal, superior mesenteric, left gastric, and celiac arteries.^[1,3]^ Arterioportal fistula (APF) is a rare vascular disorder of the abdominal viscera. They are arteriovenous communications between the splanchnic arteries and portal vein or its tributaries. APFs are more commonly intrahepatic than extrahepatic.^[4]^ Approximately 15% of APFs are caused by ruptured splanchnic artery aneurysms.^[5]^ Visceral artery pseudoaneurysm and APF occur together in rare cases. Herein, we share our experience with a unique case of acquired extrahepatic APF induced by rupture of gastroduodenal artery (GDA) pseudoaneurysm with concomitant portal vein stenosis.

## 2. Case report

**Figure 1.**
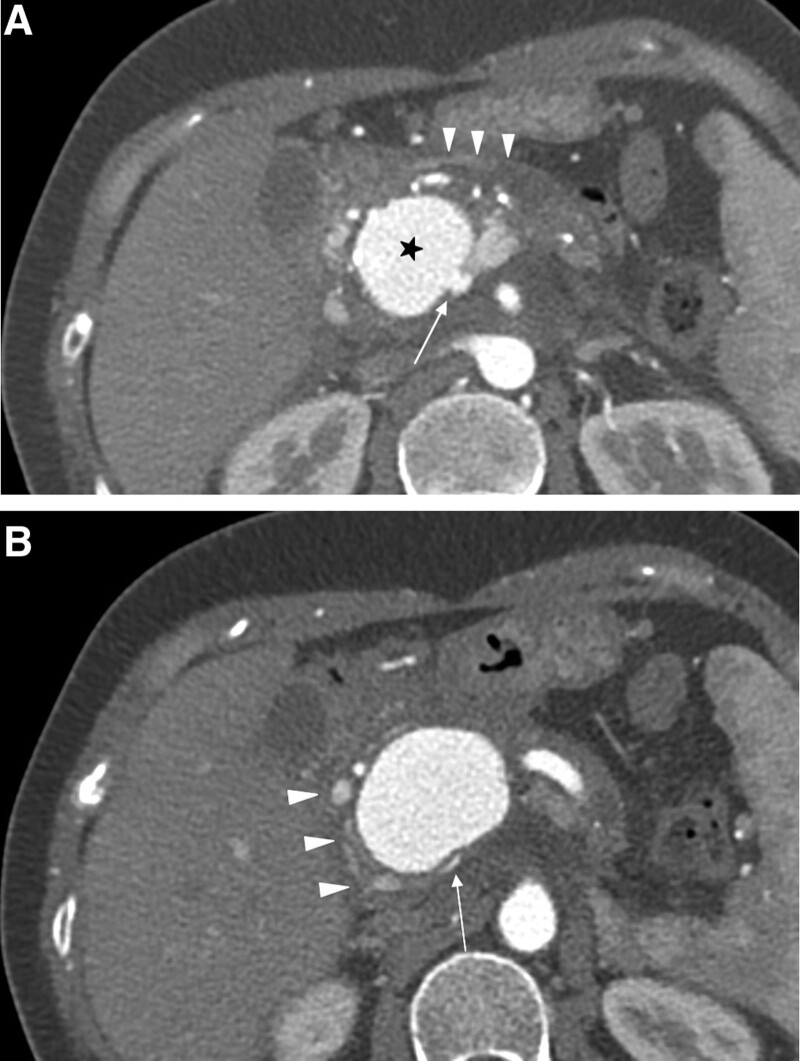
(A) Arterial phase contrast-enhanced CT scan showing GDA pseudoaneurysm (asterisk) communicating with PV (arrow). Pancreatic body shows chunky calcifications and dilatation of the main pancreatic duct (arrowheads). (B) CT scan showing PV stenosis (arrow) caused by large pseudoaneurysm, and underlying chronic pancreatitis. There is developed collateral veins (arrowheads) around GDA pseudoaneurysm. CT = computed tomography, GDA = gastroduodenal artery, PV = portal vein.

Our institutional ethical committee approved this study and informed consent was waved. A 39-years-old female with underlying chronic pancreatitis was admitted for abdominal pain and chills that started 1 week prior to presentation. She was hospitalized and discharged repeatedly because of chronic pancreatitis. She used to be alcoholic but had stopped drinking 2 years ago. Physical examination revealed mild tenderness of the lower abdomen. Laboratory tests revealed anemia (hemoglobin, 10.9 g/dL) and increased pancreatic enzyme levels (amylase, 94 U/L). Contrast-enhanced abdominal computed tomography (CT) revealed atrophy of the pancreas with parenchymal calcification and pancreatic duct dilatation. Approximately 4.5 cm-sized GDA pseudoaneurysm and portal vein stenosis with collateral vessel development were observed on the initial CT scan (Fig. 1). Because of the chronic inflammatory condition, dense adhesions and distorted anatomy were strongly suspected, moreover, portal vein stenosis was present. Therefore, it was considered that surgery would be difficult, and an endovascular treatment was decided.

**Figure 2.**
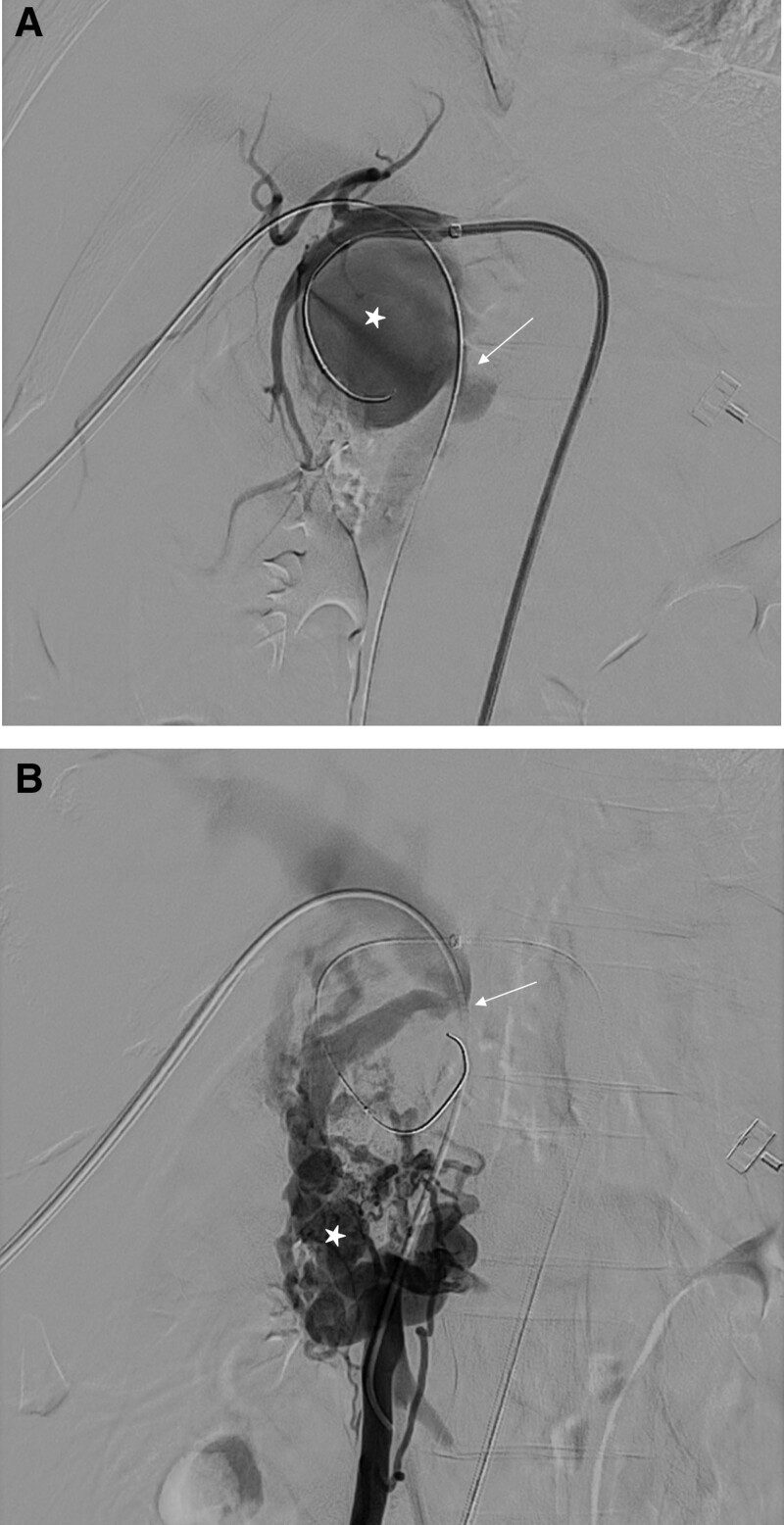
(A) Common hepatic arteriography showing GDA pseudoaneurysm (asterisk) communicating with PV (arrow). (B) Transhepatic superior mesenteric venography showing PV flow through developed collateral veins (asterisk) due to PV stenosis (arrow) and arterioportal fistula flow. GDA = gastroduodenal artery, PV = portal vein.

**Figure 3.**
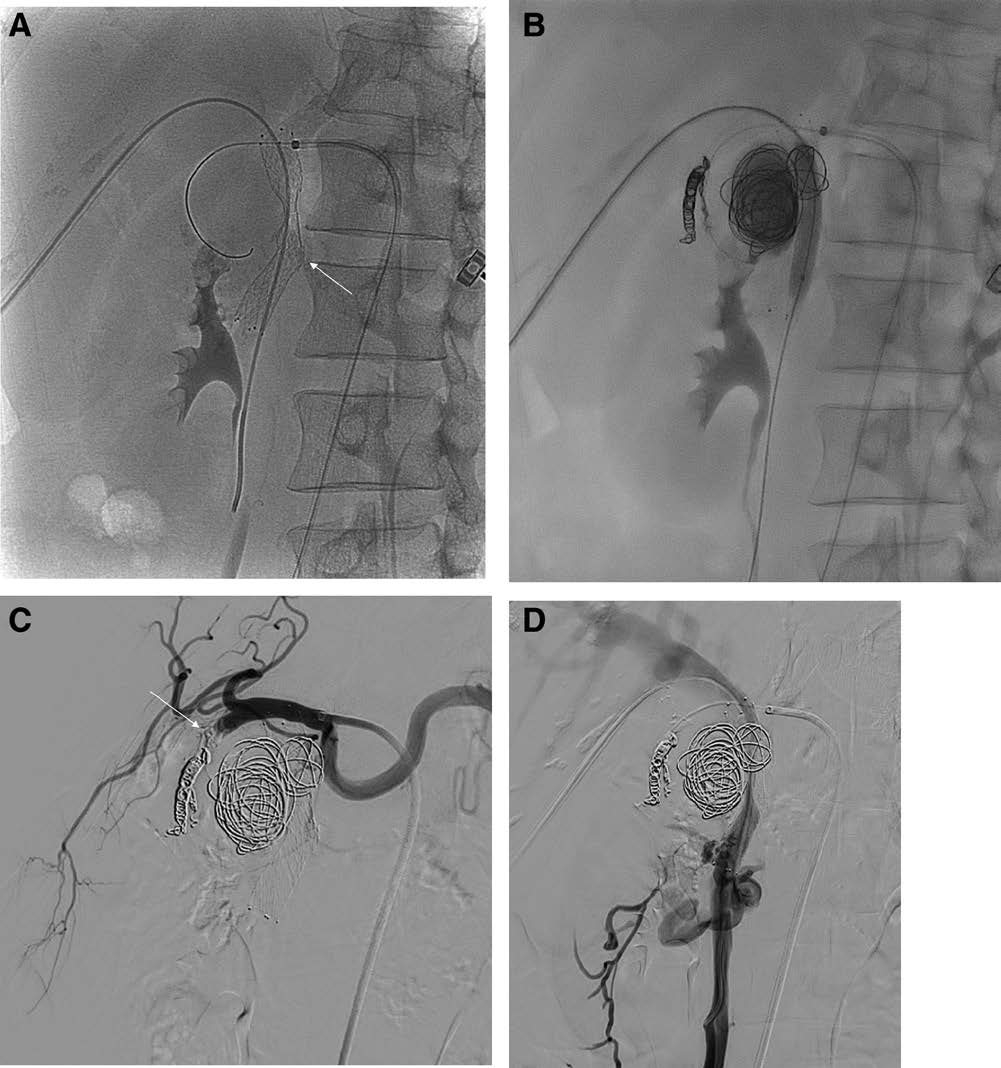
(A) Fluoroscopy reveals that PV stent (10-mm × 6-cm) (arrow) is deployed to improve PV stenosis, and prevent coil migration during coil embolization in GDA pseudoaneurysm. (B) GDA pseudoaneurysm is embolized using coils, 1:2 mixture of NBCA and lipiodol while preventing the inflow of NBCA to the PV using a balloon-assisted occlusion of the PV. (C) Common hepatic arteriography showing occluded proximal GDA (arrow) using 1:1 mixture of NBCA and lipiodol. (D) Superior mesenteric venography showing improved portal blood flow through PV stent and reduced collateral circulation. PV = portal vein, GDA = gastroduodenal artery, NBCA = N-butyl cyanoacrylate.

We punctured the right common femoral artery under ultrasound-guidance and inserted a 6F sheath. Diagnostic celiac arteriography was performed using a 5F RH catheter (Cook, Bloomington, IN). Diagnostic celiac angiography revealed a large GDA pseudoaneurysm with an APF (Fig. 2A). We also observed portal vein stenosis with developed collateral veins (Fig. 2B). The flow of superior mesenteric vein (SMV) was drained through the collateral vessels. We planned embolization of the GDA pseudoaneurysm using a trans-arterial approach and stenting for portal vein stenosis through the trans-portal approach. We replaced the 6F × 55-cm Ansel sheath (Cook, Bloomington, IN) and placed the tip of the sheath in the common hepatic artery. Using the microguide wire, 2.2F and 1.9F microcatheters (Progreat; Terumo, Japan) were passed through the pseudoaneurysm, and distal GDA. To treat portal vein stenosis, we punctured the right peripheral portal vein with the 6F sheath, and the wire was passed through the narrow area of the portal vein to the SMV using a 4F catheter (Impress diagnostic catheter; Merit Medical, South Jordan, UT). After pre-dilatation with 4-mm × 6-cm balloon (Mustang; Boston Scientific, Marlborough, MA), 10-mm × 6-cm self-expandable stent (Epic; Boston Scientific, Marlborough, MA) was deployed from proximal SMV to portal vein (Fig. 3A). We performed embolization with coils (Concerto Detachable Coil; Medtronic, Minneapolis, MN) and a 1:2 mixture of N-butyl cyanoacrylate (NBCA), and lipiodol through the 2.2F microcatheter. Before using the mixture of NBCA and lipiodol, we inflated an 8-mm × 4-cm balloon catheter (Mustang; Boston Scientific, Marlborough, MA) in the portal vein to prevent leakage of the NBCA and lipiodol (Fig. 3B). The proximal and distal GDA and superior pancreaticoduodenal artery were occluded with coils, and a 1:1 mixture of NBCA and lipiodol using the sandwich method (upstream and downstream occlusion) through the 1.9F microcatheter.

The final angiogram indicated complete obliteration of the pseudoaneurysm (Fig. 3C). On the mesenteric venogram, it was confirmed that the drain through the collateral vein disappeared, and the flow drain to the portal vein stent was restored (Fig. 3D). The patient reported the remission of abdominal pain after endovascular management, and her hemoglobin level returned to normal (hemoglobin, 13.1 g/dL) after transfusion with 2 units of packed red blood cells. The patient recovered without any complications and was discharged. CT performed after 1 month revealed an embolized GDA pseudoaneurysm, and a patent stent in the portal vein.

## 3. Discussion

Chronic pancreatitis is an ongoing fibroinflammatory disease of the pancreas characterized by irreversible damage to the pancreatic parenchyma and ductal system. As chronic pancreatitis progresses, various symptoms and complications occur. Clinically, the following symptoms occur: abdominal pain, and pancreatic insufficiency (endocrine or exocrine). Moreover, the following diseases may occur: metabolic bone diseases, pseudocysts, pancreatic adenocarcinoma, biliary obstruction, and vascular complications ranging from venous thrombosis to rare but life-threatening bleeding pseudoaneurysms.^[6,7]^ Visceral artery pseudoaneurysms are uncommon sequelae of pancreatitis, either acute or chronic. In pancreatitis, pseudoaneurysm is caused by erosion of nearby vessels due to digestive enzyme leakage.^[1]^ As mentioned earlier, it is most common in the splenic artery and relatively rare in the GDA.

APFs are a complex group of arteriovenous fistulas with arteriovenous communication between the splanchnic arteries, and portal vein or its tributaries. They can be acquired or congenital. The most common etiology of APF is trauma (e.g., blunt, stab gunshot wounds) followed by iatrogenic causes such as surgical and non-surgical interventions, congenital abnormalities, neoplasms, and rupture of pseudoaneuryms.^[4,5]^ Pseudoaneurysm and APFs are associated with conditions ranging from asymptomatic to life-threatening, such as peritoneal or retroperitoneal hemorrhage.^[6]^ Besides, APFs have large shunt flow, and are therefore likely to cause symptoms of portal hypertension.^[4]^

The diagnosis of complications of chronic pancreatitis, including pseudoaneurysm and APFs, are possible with various modalities, such as contrast-enhanced CT, magnetic resonance imaging, ultrasonography with Doppler, or angiography. Contrast-enhanced CT is useful for detecting small aneurysms, and assessing anatomical details by providing optimal visualization of these vascular abnormalities. Our patient was also initially confirmed using contrast-enhanced CT, and was diagnosed with acquired extrahepatic APFs induced by the rupture of a GDA pseudoaneurysm. Portal vein stenosis with collateral vessel development was also observed.

In chronic pancreatitis, an ongoing fibroinflammatory disease, scarring, or compression by pseudocysts may affect the peripancreatic veins. As a result, portal and splenic vein narrowing or venous thrombosis may occur.^[8]^ Therefore, all these lesions appear to be eventually caused by chronic pancreatitis. However, in this case, it was not possible to determine whether the cause of portal vein stenosis was chronic pancreatitis or compression by a large GDA pseudoaneurysm.

To review the available English language literature, a systematic search was conducted from the 2 databases PubMed, and Google Scholar using a combination of the following key words: “Chronic pancreatitis,” “Pancreatitis,” “Arterioportal fistula; APF,” “Pseudoaneurysm,” “Splanchnic artery,” “Gastroduodenal artery; GDA,” “Portal vein,” “Stenosis” and “Compression.” As a result of the search, there were cases of obstructive jaundice due to compression of the common bile duct by GDA pseudoaneurysm,^[2]^ celiac axis stenosis due to true pancreatoduodenal arcade aneurysm,^[9]^ portal vein stenosis due to pseudoaneurysm of the hepatic and gastroduodenal arteries,^[10]^ hepatic artery pseudoaneurysm, and portal vein stenosis due to inflammatory changes that occurred after a leak at the hepaticojejunostomy,^[11]^ portal vein thrombosis and stenosis with cavernous transformation due to chronic pancreatitis,^[12,13]^ and APF due to a ruptured pancreaticoduodenal pseudoaneurysm.^[4]^ There was a case similar to ours, in which GDA pseudoaneurysm and APF coexisted in chronic pancreatitis^[14]^; however, portal vein stenosis did not exist as in our case.

Endovascular treatment has recently become mainstream for the management of pseudoaneurysms. However, the appropriate treatment depends on the patient’s condition. Prior to treatment, vessel anatomy, location, etiology of disease, and the patient’s underlying condition must be considered when deciding whether to choose endovascular or surgical therapy.^[15]^ If the patient’s vital signs are unstable, it is an indication for surgical treatment; however, surgical access is sometimes difficult. In cases where the injured vessel usually lies deep within the pancreatic parenchyma or pseudocyst, it is difficult to accurately define the exact location.^[16]^ Additionally, dense adhesions and distorted anatomy caused by long-standing inflammation may complicate surgical treatment.^[17]^

In our case, we chose interventional endovascular treatment because surgical treatment was difficult owing to the high suspicion of chronic inflammatory changes as described above, and concomitant portal vein stenosis. We performed a combined trans-arterial embolization (TAE) and transportal stenting. We embolized the inflow of the pseudoaneurysm using TAE, and the outflow from the APF was blocked using a stent and balloon catheter to prevent the microcoils and NBCA from flowing into the portal vein. Because 1 report described a case in which the coil for TAE flowed to the portal vein through the APF,^[18]^ it seems important to back up the portal vein side with a balloon. Since we used NBCA concomitantly we think ballooning of the portal side is more important to prevent NBCA from exiting the fistula. In our case, the portal vein stent served 2 roles: preventing the migration of coils during TAE and improving portal vein stenosis. As a result, portal vein flow was restored, and the blood flow of the GDA pseudoaneurysm was completely controlled.

Owing to the developments in endovascular treatment, it is being used to treat various complications of chronic pancreatitis. However, although treatments for pseudoaneurysm, portal vein stenosis, or APF have been widely used, such a complex case is unique. Nevertheless, if a treatment plan is well established according to the patient’s condition and CT, it will be possible to successfully treat even a complex case such as ours.

## 4. Conclusion

This is an extremely rare case of simultaneous occurrence of GDA pseudoaneurysm, APF, and portal vein stenosis caused by chronic pancreatitis. Chronic pancreatitis can present with a variety of complications and is sometimes life threatening. Occasionally, long-term inflammatory changes may make it difficult to determine the treatment options. However, combined TAE and transportal stenting can be helpful.

## Acknowledgments

The authors would like to thank “Editage Language Editing Service” for the English language review and editing; http://www.editage.co.kr/.

## Author contributions

**Conceptualization:** Sung Eun Park, Jae Yool Jang.

**Data curation:** Sung Gong Lim, Sung Eun Park, Jae Yool Jang.

**Formal analysis:** Jung Ho Won, Jae Yool Jang.

**Investigation:** In Chul Nam, Eun Cho.

**Methodology:** Ho Cheol Choi, Sa Hong Jo.

**Supervision:** Sung Eun Park, Ho Cheol Choi, Hye Jin Baek.

**Validation:** In Chul Nam, Sa Hong Jo, Jin Il Moon, Eun Cho, Jae Yool Jang.

**Visualization:** Jung Ho Won, Sa Hong Jo.

**Writing – original draft:** Sung Gong Lim.

**Writing – review & editing:** Sung Eun Park, Hye Jin Baek.
